# Microvascular Decompression for Trigeminal Neuralgia Secondary to Vertebrobasilar Dolichoectasia: Review of the Literature and Illustrative Case

**DOI:** 10.3390/jcm13216342

**Published:** 2024-10-23

**Authors:** Massimiliano Visocchi, Fabio Zeoli, Francesco Signorelli

**Affiliations:** 1 Institute of Neurosurgery, Fondazione Policlinico Universitario A. Gemelli IRCCS, Catholic University, 00136 Rome, Italy; mvisocchi@hotmail.com (M.V.); francesco.signorelli@policlinicogemelli.it (F.S.); 2 Research Center and Master II Degree Surgical Approaches Craniovertebral Junction, Fondazione Policlinico Universitario A. Gemelli IRCCS, Catholic University, 00136 Rome, Italy

**Keywords:** microvascular decompression, vertebrobasilar dolichoectasia, trigeminal neuralgia

## Abstract

Classical trigeminal neuralgia (TN) is a chronic pain disorder characterized by severe, unilateral facial pain, often resulting from vasculonervous conflict. A less common cause of TN is vertebrobasilar dolichoectasia (VBD). Microvascular decompression (MVD) is the preferred surgical intervention for TN, but in case of VBD, the surgical procedure is more complex due to the aberrant vascular anatomy. This study aims to review the evidence on MVD for VBD-induced TN, and analyze operative challenges, efficacy, and postoperative outcomes. An illustrative case is also presented. We report on the case of a 62-year-old male with a 7-year history of right-sided TN affecting the maxillary (V2) and mandibular (V3) territories. The patient underwent MVD using the interposition technique, where Teflon sponges were placed between the basilar artery and the nerve. Postoperatively, the patient experienced complete pain relief without neurological deficits. At 12 months follow-up, the patient remained pain-free and off medications. We performed an extensive literature review using PubMed, Scopus, and Web of Sciences, highlighting the most relevant studies and findings on the topic. The literature review showed that, while MVD is effective in providing long-term pain relief in VBD-induced TN, the choice between interposition and transposition techniques remains debated. Interposition is easier to perform but may inadvertently increase nerve compression in some cases, whereas transposition offers more definitive decompression but carries higher technical risks. Our case and the available literature highlight the importance of individualized treatment planning in achieving optimal outcomes for patients with VBD-induced TN. Further research is needed to refine surgical techniques and minimize complications in this subset of TN patients.

## 1. Introduction

Classical Trigeminal Neuralgia (TN) due to vasculonervous conflict is a common chronic pain disorder characterized by recurrent episodes of electric shock-like or stabbing pain affecting the dermatomal distribution of trigeminal nerve [1,2,3]. This condition is almost always unilateral [4]; it manifests more commonly on the left side and within the territories of the maxillary (V2) and mandibular (V3) branches of the trigeminal nerve [5,6]. The incidence of TN is higher in women and increases with age [7]. The most implicated blood vessels responsible for compression of the trigeminal root are the superior cerebellar artery, in about 75% of cases, the anterior inferior cerebellar artery, in 10%, or a vein, in 7% [3,8]. Less commonly, TN may be secondary to vertebrobasilar dolichoectasia (VBD). VBD, as originally described by Smoker et al., is a rare cerebrovascular disease characterized by ectatic, elongated, and tortuous vertebrobasilar arteries (VBA) [9]. The abnormal arteries may sometimes compress, directly or indirectly—thorough displacements of adjacent vessels [7]—the root of the trigeminal nerve, resulting in TN [10]. Although still largely unknown, researchers have associated VBD pathogenesis to a combination of congenital factors (defect of the arterial elastic layer) and acquired vascular factors such as hypertension and atherosclerosis [7,11,12,13].

Accurate diagnosis of trigeminal nerve compression is a critical step before considering surgical treatment. This is typically achieved using brain MRI, including 3D-CISS (T2-weighted), 3D-TOF, or contrast-enhanced angio-MRI (T1-weighted). The 3D-FIESTA sequence provides high-resolution images of the cranial nerves and vascular structures in contrast with cerebrospinal fluid (CSF), while the other sequences show vascular structures in hypersignal, allowing for clear differentiation with cranial nerves. Additionally, a recent study [14] has demonstrated the utility of arterial spin labeling (ASL) MRI, a dedicated sequence capable of accurately detecting complex vascular malformations.

MVD is currently proposed as the first surgical choice for the treatment of TN [6,7,15,16]. Several variations of decompression procedures for prominent offending arteries have been proposed over the past two decades. In VBD cases, given the unusual anatomy and the low elasticity of the atherosclerotic vertebrobasilar artery, surgery is highly challenging and requires considerable experience and dexterity.

Our work aims to review the available evidence on MVD for trigeminal neuralgia secondary to VBD, including a case from our institution, and analyze surgical strategies, efficacy, and rate of postoperative complications.

## 2. Case Presentation

A 62-year-old male patient presented to our department with a seven-year history of paroxysmal, lancinating right-sided trigeminal neuralgia in maxillary (V2) and mandibular (V3) territories. The pain was described as sharp and electrical-like and was triggered by talking and chewing. Over time, the pain significantly worsened in terms of frequency and intensity of episodes, particularly in the four months before admission. The pain was not satisfactorily controlled by oral opioids, tricyclic, or dual antidepressants (Tapentadol 100 mg/day, Carbamazepine 400 mg/day) and worsened every time the treatment was scaled down (Grade V pain according to the Barrow Neurological Institute grading system, Table 1 [2]).

**Figure 1:**
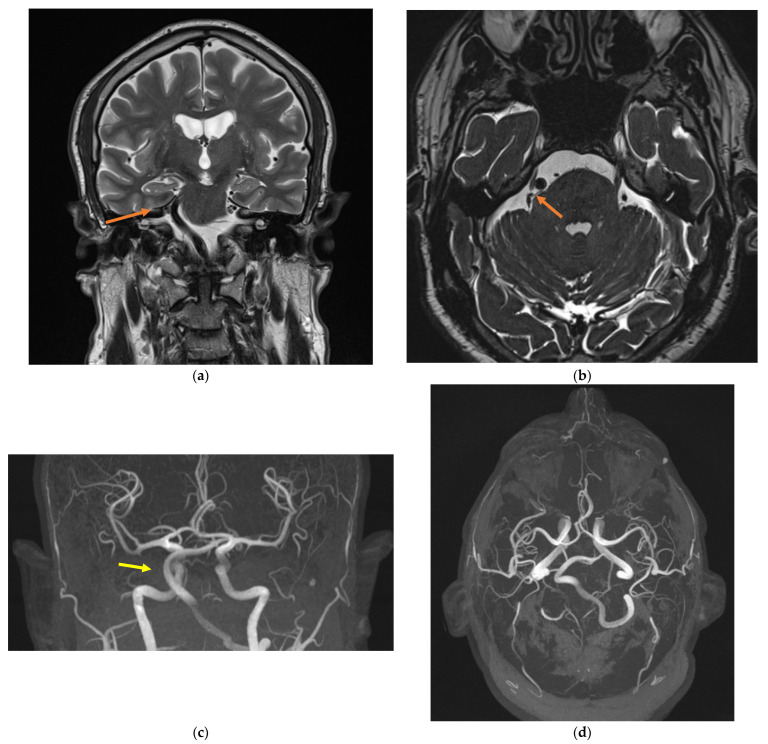
Magnetic resonance imaging showing the neurovascular conflict (**a**,**b**; orange arrow) and the vertebrobasilar dolichoectasia (**c**,**d**; yellow arrow). (**a**): coronal plane, T2-weighted sequence; (**b**): axial plane, 3D-CISS sequence; (**c**): coronal plane, MRI angiogram; (**d**): sagittal plan, MRI angiogram.

The patient’s past medical history included bilateral high-frequency hearing loss, bronchial asthma, essential hypertension, and myocardial infarction treated with percutaneous coronary intervention and double antiplatelet therapy. On examination, the motor and sensory functions of the trigeminal nerve and corneal reflex were intact; no neurological deficit was found. Routine blood tests (metabolic panel and complete blood count) were normal. Brain magnetic resonance imaging (MRI) with gadolinium contrast revealed the presence of an abnormality of the right vertebral artery (VA) (no visualization of V4 segment), with ectasia of the left VA, and a dolichoectatic basilar artery (BA) with a tortuous course causing, at the right cerebellopontine angle (CPA), a mechanical compression on the trigeminal nerve (Figure 1). The compression of the TN was grade II according to Sindou et al. classification [17] (Table 2).

Considering the clinical and radiological findings, in addition to low tolerance and ineffectiveness of medical therapy, microvascular decompression surgery was proposed.

A right retrosigmoid approach was performed under general anesthesia, with the patient placed in a left park bench position. After reaching the trigeminal cistern, we opened the dura and drained the cerebrospinal fluid (CSF). Following the release of CSF, the cerebellum was retracted medially, exposing the CPA and trigeminal nerve. Arachnoid dissection revealed the dolichoectatic BA compressing the right anterolateral region of the brainstem and the trigeminal nerve root. Microvascular decompression of the dolichoectatic BA was obtained using the interposition technique: two layers of Teflon sponge, held with fibrin glue, were placed between the artery and the nerve. Particular care was taken to avoid injury of the artery during the vascular microdissection, given the potentially fragile vessel walls in VBD. The Dandy vein was preserved.

**Figure 2:**
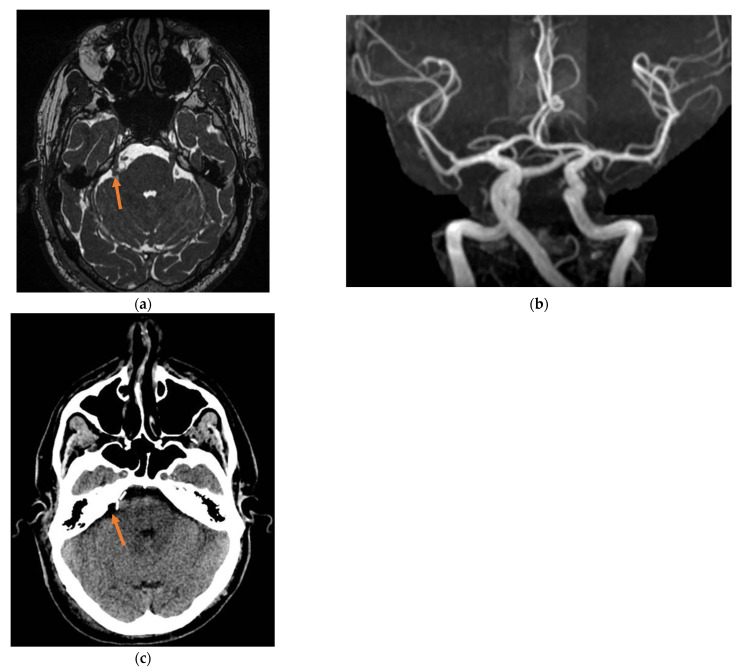
Post-surgery imaging showing Teflon sponges placed between the dolichoectatic basilar artery and the right trigeminal root (orange arrow). (**a**): MRI, axial plane, (**b**): MRI Angiogram, (**c**): CT scan, axial plane.

Pain completely resolved after surgery and no neurological deficits were reported. Postoperative brain CT scan and MRI (Figure 2) showed satisfactory surgical outcomes and excluded bleeding complications. The patient was discharged on day 6 after surgery. The patient reported satisfactory pain control (BNI grade 1) at 1-month follow-up. Four weeks after surgery, medications were completely and definitively stopped. No pain recurrence (BNI grade I) and/or sensory abnormalities were found at 3/6/12 months follow-up, and the patient is still on a medication-free status.

## 3. Discussion

Vertebrobasilar dolichoectasia (VBD; from the Greek *dolicho*, “elongated,” and *ectasia*, “dilated”) is an uncommon cause of trigeminal neuralgia (TN). Previous reports on this topic are limited. We conducted an extensive search of the literature and collected the most relevant studies on VBA in TN. A summary of the main findings is reported in Table 3.

VBD refers to a vascular abnormality characterized by expansion, elongation, and tortuosity of the vertebrobasilar system. Diagnostic criteria, first proposed by Ubogu et al. [18], include basilar artery (BA) or vertebral artery (VA) diameter > 4.5 mm, deviation of any portion >10 mm from the shortest expected course, BA length > 29.5 mm or intracranial VA length > 23.5 mm; also, for the BA, when bifurcation is above the suprasellar cistern or if any portion lies adjacent to the margin of the clivus or dorsum sellae, elongation is considered to be present [19].

### 3.1. Pathophysiology, Clinical Characteristics, and Medical Treatment

Although sometimes asymptomatic, VBD can be associated with posterior circulation transient ischemic attacks and strokes [18,20,21,22], subarachnoid bleeding [23], brainstem compression [24], hemifacial spasm [25,26], and trigeminal neuralgia [27]. According to previous studies, VBD-induced TN accounts for 2% to 7.7% of all cases of TN [3,13,28,29,30]. Although the etiopathogenesis of VBD is still largely unknown, most of the evidence points towards a multifactorial process combining congenital vascular wall abnormalities with acquired factors related to atherosclerosis [31,32]. Compared to other patients affected by TN, patients with VBD-related TN are more likely to be older, male, and with a history of diabetes, hyperlipidemia, hypertension, and myocardial infarction [10,13,16,33,34,35].

The elongation and tortuosity of the VBA system can dislocate adjacent vessels, which consequently reach the trigeminal nerve and contribute to compression. Among these, the anterior inferior cerebellar artery (AICA) is the most common additional vessel found in TN cases secondary to VBD [6,10]. The involvement of multiple vessels necessitates a thorough surgical approach, as all compressive forces must be addressed to relieve the trigeminal nerve effectively. In TN secondary to VBD, pain occurs more commonly, but not exclusively, as our case shows, on the left side, and left predominance may be attributed to the asymmetry in the caliber of the two vertebral arteries that commonly occurs or a stronger transmission of pulse pressure to the left VA from a subclavian artery that has its origin from the aortic arch rather than the brachiocephalic trunk. [10,27,33,36]. Most commonly, patients complain of pain in the V2 and/or V3 dermatome. As with other forms of classical TN, medical therapy—primarily with carbamazepine [37] or oxcarbazepine—remains the first-line treatment in VBD-associated TN. When pharmacological therapy proves ineffective or is not well tolerated, surgical options such as trigeminal rhizotomy, percutaneous thermocoagulation, mechanical balloon compression, microvascular decompression (MVD), and gamma knife radiosurgery can be considered [13,35].

### 3.2. Surgical Management

Microvascular decompression (MVD) has demonstrated the highest efficacy among surgical treatments for VBD-related TN [38,39,40,41,42,43], as it is nondestructive and directly addresses the vascular etiology. Historically, the retrosigmoid approach has been the standard method to reach the trigeminal nerve at the root entry zone; however, in recent years, the endoscopic transorbital approach (ETOA) has emerged as a viable, minimally invasive, surgical option, particularly for treating Schwannomas of the trigeminal nerve and tumors affecting the middle cranial fossa [44,45]. MVD, first described by Jannetta et al. in 1967 [40], aims at isolating the trigeminal nerve from the vessel responsible for compression by placing a piece of material between the vessel and the nerve. When the artery involved is a dolichoectatic VBA, the surgery is notably more challenging; dolichoectatic arteries present with atherosclerosis, abnormal course, increased diameter, low elasticity, and limited mobility. These factors significantly amplify the complexity of the surgery, particularly during vessel displacement, posing potential risks such as plaque dislodgement, injury to brainstem perforators, and vasospasm [36]. Several studies have demonstrated the superiority of MVD over alternative surgical procedures in ensuring long-term pain relief with a low complication rate [3,38,46,47]. However, reports on the efficacy and safety of MVD specifically in cases involving VBD are scarce. Di Carlo et al. [27] conducted a random-effects meta-analysis, including 167 patients with TN secondary to VBD, and found that 97% of cases experienced immediate postoperative pain relief, with a slight decrease to 92.9%, at the last follow-up (55 months). Early post-operative complications included facial numbness (21% of cases), diplopia (5%), hearing impairment (5%), and cerebellar ataxia (4%). By the last follow-up, hearing impairment remained stable (5%), while rates of facial numbness, diplopia, and cerebellar ataxia decreased to 13%, 0%, and 3%, respectively. In a cohort study by Zheng et al. [7], no significant differences were found in the safety and efficacy of MVD between the VBD group and the conventional TN group (*p* > 0.05). However, some authors have reported higher failure or recurrence rates in patients with elongated, ectatic VBA treated with MVD [33,48,49].

### 3.3. Interposition vs. Transposition

Various techniques have been applied during MVD to separate the offending dolichoectatic VBA from the compressed trigeminal nerve. These can be broadly classified into the interposition method and transposition method [7,13,35]. The former, which is the traditional method and is widely used in patients with neurovascular compression syndrome due to small vessels such as the SCA and AICA, involves the insertion of an implant between the offending vessel and the nerve [3,10,16,30,36,50]; the latter method involves repositioning the VBA using biomedical glue [51], Teflon slings or pads [28,32,52], and aneurysm clips [53]. Both methods have advantages and disadvantages.

The conventional interposition technique is widely accepted as it is easy to perform, can be effectively achieved through a relatively small craniotomy, and requires minimal manipulation of the VBA complex. However, some authors argue that, in cases of VBD, this technique may worsen the symptoms as the offending VBA follows a prominent upward loop squeezing the CN V against the cerebellar tentorium; therefore, inserting material between the artery and the nerve may ultimately increase the compression effect [6,7,52]. Despite these concerns, several case series have reported successful long-term outcomes with the interposition in VBD cases [3,10,16,28,30,36,50].

The transposition technique has been proposed as an effective and safe alternative to interposition [13]. It relieves the pressure on the nerves by repositioning the VBA—thereby eliminating the possibility of indirect compression—using materials such as biomedical glue, Teflon, and aneurysm clips. This approach is not free of risks. When trying to mobilize the VBA, the following factors must be considered: (i) the VBA complex is stiff, tortuous, and ectatic, making it difficult to dislocate; (ii) overstretching and injury of the perforator branches originating from VBA can result in brainstem infarction; (iii) atherosclerotic plaques have the potential to break during mobilization, which can serve as a source of emboli and may cause ischemic stroke or cerebral hemorrhage [51]. Chai et al. [13] found that the 5-year rate of pain relief (BNI I) for the transposition technique was 91.1%; this was compared to 58.7% for the interposition technique, with no significant difference in terms of postoperative complication rate. Inoue et al. [6] decompressed the trigeminal nerve by displacing the VBA and found a long-term pain relief rate of 96%. Concerning the adequate and secure distancing of the VBA from CN V needed to ensure effective decompression, different theories have been proposed. According to Amagasaki et al. [52], the surgeon must mobilize the VBA as caudally as possible; this is because the trigeminal nerve is often shifted rostrally by the VBA, and after repositioning the vessel, CN V often droops and may contact the VBA again. However, according to other authors, such extensive mobilization dramatically increases surgical times and may result in kinking and stenosis of the parent artery, overstretching and injury to the perforating arteries, and brain stem infarction [53]. Also, it does not significantly impact long-term pain relief, as shown in the study by Inoue et al. [6]: they compared the “separation group”—where complete separation of the VBA from the trigeminal nerve was achieved—and the “contact group”—where slight vascular contact remained—and found no difference in terms of pain relief, indicating that once the tense trigeminal nerve is loosened, further attempts to mobilize the VBA for complete nerve separation are not necessary. Several surgeons prefer to mobilize the VBA using Teflon felts placed piece by piece between the vessel and surrounding structures [6,7,32,54]. However, large volumes of Teflon increase the risk of inflammation, granuloma formation, and adhesions, ultimately resulting in TN recurrence. In the case series by Wang et al. [54], including patients treated with VBA repositioning using Teflon felts, one patient developed recurrent post-operative pain that required a second surgery to remove the redundant Teflon and adhesions. Techniques that involve fixing the transposed VBA to the skull base dura (at the clivus or petrous region) have been developed to secure the repositioning. Liu et al. [51] successfully treated 22 cases of VBD-induced TN by mobilizing the VBA with Teflon pads and fixing it to the petrous dura using biomedical glue. A similar technique was used in previous smaller case series, with comparable outcomes reported [32,35]. Although relatively simple to perform, glue fixation suffers several limitations. Above all, the adhesive is not strong enough to guarantee a permanent fixation. An effective solution to overcome this problem is to coagulate the portion of the dura where the artery will be fixed to induce a scar to promote fibrous adhesion [35,51,53]; pieces of Surgicel may also be used [32]. Additionally, the use of glue poses the potential risk of inducing vasculitis and cranial nerve damage from glue overflow or dripping. Also, recurrence due to the adhesion of neurovascular structures by the glue has been reported in the literature [26,55].

A safer alternative to glue fixation is the use of aneurysm clips, as described by Lin et al. in 2012. [53] They reported a series of three cases of VBD-induced TN successfully treated by transposing the offending vessel using an unabsorbable dural tape sling and fixing it with an aneurysm clip passed through a petrous dural bridge. Besides ensuring effective and durable fixation, this technique has the major advantage of allowing an easy modulation of the strength of traction (the more of the sling the clip takes, the stronger the traction) and the direction of transposition, thus avoiding kinking of the parent artery, overstretching of the perforating branches, and damaging the adjacent cranial nerves. However, when considering the fixation technique, it is worth noting that placing vascular clips introduces radiological artifacts that may affect later imaging evaluation.

Although MVD, whether through the interposition or transposition method, has proven highly successful in cases of VBD, significant postoperative complications can arise. Notably, diplopia and hearing loss are relatively common, due to mechanical stimulation of the fourth, sixth, or eighth cranial nerves during decompression, especially in cases of extensive manipulation of the VBA complex [13,35].

Our study has some limitations. The included articles were primarily small, retrospective, single-institution case series, and many reported incomplete data. Follow-up ranged from 1 year up to 15 years, which may not provide a homogeneous long-term perspective. Future research should prioritize larger, prospective studies with standardized reporting to enable better comparison of surgical techniques and strategies.

**Table 3:** Summary of the studies included in the literature review.

Authors	Title	Publication Year	Study Design	Sample Size (M/F, Mean Age)	Type of Intervention	Operative Technique	Outcomes	Main Findings
Zheng et al. [7]	Trigeminal neuralgia caused by vertebrobasilar dolichoectasia: efficacy of stepwise decompression technique	2023	Cohort study	61 (31/30, 59.8 y.o.)	VBA transposition (Stepwise decompression)	VBA complex was repositioned by gradually placing Teflon felts from the proximal end of the VBA to the point of vascular force, with each felt acting as a “bridge pier”	Efficacy (BNI grade I-III); pain recurrence rate (reappearance of pain or aggravation after pain relief; time > 6 months); postoperative complications rate.	Complete pain relief was achieved in 93.4% of cases; 8.2% of cases reported pain recurrence at last follow-up; most reported postoperative complications included: facial numbness (11.5%), facial palsy (8.2%), and hearing impairment (4.9%)
Amagasaki et al. [52]	Macrovascular Decompression with the Transposition Method Using Teflon Sling for Trigeminal Neuralgia Caused by the Vertebrobasilar Artery	2022	Case series	32 (12/20, 66.3 y.o.)	VBA Transposition	A Teflon sling was applied directly to the trunk of the VBA complex, and then pulled toward the lower clivus region	Efficacy (BNI score); pain recurrence rate; duration of surgery; postoperative complications rate.	Complete pain relief was achieved in 93% of cases; 1 patient reported pain recurrence; average surgical time was 290 min; permanent cranial nerves (VI, VIII) disorders were observed in 21% of cases.
Inoue et al. [6]	Microvascular decompression for trigeminal neuralgia attributable to the vertebrobasilar artery: decompression technique and significance of separation from the nerve root	2021	Case series	26 (13/13, 68 y.o.)	VBA Transposition	A roll-shaped Teflon was placed to relocate the VBA complex away from the nerve root. Complete separation was obtained in 13 cases (the separation group), whereas slight contact with the nerve remained in the remaining 13 cases (the contact group).	Efficacy; pain recurrence rate; postoperative complications rate.	Pain relief was achieved in 100% of cases; 3.4% of cases reported pain recurrence at the last follow-up; postoperatively 19.2% of cases reported facial numbness, and 8% hearing impairment.
Wang et al. [54]	The long-term clinical outcomes of microvascular decompression for treatment of trigeminal neuralgia compressed by the vertebra-basilar artery: a case series review	2019	Case series	23 (9/14, 62.8 y.o.)	VBA Transposition	Roll-shaped Teflon implants were placed piece by piece between the VBA complex and the brain stem, gradually pushing the large vessels close to the skull base and away from the nerve root. Additional Teflon materials were implanted between the nerve and the vessels to prevent the vascular loop from rebounding.	Efficacy; pain recurrence rate; postoperative complications rate.	Pain relief was achieved in 96% of cases; 13% of cases reported pain recurrence at last follow-up; most reported postoperative complications included: facial numbness (17%), diplopia (17%), hearing impairment (4.3%)
Liu et al. [51]	Biomedical Glue Sling Technique in Microvascular Decompression for Trigeminal Neuralgia Caused by Atherosclerotic Vertebrobasilar Artery: A Description of Operative Technique and Clinical Outcomes	2019	Case series	22 (9/13, 63 y.o.)	VBA Transposition	VBA complex was transposed from the trigeminal nerve using Teflon pads and then fixed on the coagulated petrous dura with biomedical glue.	Efficacy; pain recurrence rate; postoperative complications rate.	Complete pain relief was achieved in 100% of cases; pain recurrence was reported in 9% of cases; 4.5% of cases reported postoperative hearing impairment.
Vanaclocha et al. [35]	Is There a Safe and Effective Way to Treat Trigeminal Neuralgia Associated with Vertebrobasilar Dolichoectasia? Presentation of 8 Cases and Literature Review	2016	Case series	8 (5/3, 64.9 y.o.)	VBA Transposition	VBA complex was transposed medially towards a portion of coagulated clivus dura and supported in place with Teflon implants. In 3 cases, human fibrin glue was added to hold the VBD in its new position	Efficacy; pain recurrence rate; postoperative complications rate.	Pain relief was achieved in 100% of cases; no recurrence was reported at last follow-up; 12.5% of cases reported hearing impairment.
Lin et al. [53]	An easy adjustable method of ectatic vertebrobasilar artery transposition for microvascular decompression	2012	Case series	3 (1/2, 73 y.o.)	VBA Transposition	VBA complex was transposed using a sling of unabsorbable dural tape, which was fixed to the dura over the petrous bone with an aneurysm clip through a dural bridge.	Efficacy; pain recurrence rate; postoperative complications rate.	Pain relief was achieved in 100% of cases; non recurrence was reported at last ollow-up;
Yang et al. [28]	Microvascular decompression on patients with trigeminal neuralgia caused by ectatic vertebrobasilar artery complex: technique notes	2012	Case series	10 (5/5, 64 y.o.)	VBA Transposition	The VBA complex was transposed caudolaterally by gradually placing shredded Teflon sponge between the artery and the brainstem.	Efficacy; pain recurrence rate; postoperative complications rate.	Complete pain relief was achieved in 100% of cases; no patients reported pain recurrence at the last follow-up; 10% patients reported postoperative facial numbness.
Yamahata et a. [32]	Microvascular Decompression for Trigeminal Neuralgia due to Compression by the Vertebral Artery: Report of 3 Cases	2011	Case series	3 (1/2, 71.3 y.o.)	VBA Transposition	VBA complex was transposed using Teflon slings and then fixed to the clival dura with fibrin glue.	Efficacy; pain recurrence rate.	All patients experienced complete pain relief with no recurrence reported at the last follow-up
Linskey et al. [33]	Microvascular decompression for trigeminal neuralgia caused by vertebrobasilar compression	1994	Cohort study	31 (21/10, 62 y.o.)	VBA Transposition	VBA complex was transposed and held in position using cushions of Teflon felt, Ivalon sponge, autologous muscle, or silicon sheet; in one case the ectatic vertebral artery was held away from the trigeminal nerve by passing the vessel through the fenestration of a straight aneurysm clip and suturing the clip to the tentorium.	Efficacy; pain recurrence rate; postoperative complications rate.	Postoperative complete pain relief was achieved in 100% of cases; 3 patients (9.6%) reported pain recurrence at the last follow-up; transient diplopia due to either trochlear or abducens nerve paresis was reported in 22.6% of cases, and hearing loss occurred in 12.9%.
Chai et al. [13]	Microvascular decompression for trigeminal neuralgia caused by vertebrobasilar dolichoectasia: interposition technique vs. transposition technique	2020	Cohort study	39 (24/15, 61.4 y.o.)	Interposition (*n* = 16); VBA Transposition (*n* = 23)	INTERPOSITION group: pieces of Teflon or gelatin pledges were placed in the neurovascular conflicting area between the VBA complex and the trigeminal nerve. TRANSPOSITION group: the VBA complex was transposed using Teflon slings and then fixed to the coagulated petrous dura with α-cyanoacrylate glue.	Efficacy; pain recurrence rate; postoperative complications rate.	INTERPOSITION group: postoperative complete pain relief (BNI I) was reported in 87.5% of cases; 25% of patients reported pain recurrence at last follow-up; postoperative complications included: facial palsy (6.25%), facial hypoesthesia (18.75), hearing loss (6.25%); TRANSPOSITION group: postoperative complete pain relief (BNI I) was reported in 100% of cases; 4% of patients (1 out of 23) reported pain recurrence at last follow-up; postoperative complications included: taste hypoesthesia (4%), hearing loss (8.6%), diplopia (4.35%). was similar in both groups. At the last follow-up, the transposition group showed significantly better long-term BNI scores compared with the interposition group (*p* = 0.03); incidence of postoperative complications was similar be-ween the two groups.
Honey and Kaufmann [15]	Trigeminal Neuralgia due to Vertebrobasilar Artery Compression	2018	Case series	13 (10/3, 67.3 y.o.)	Interposition (n = 2); VBA Transposition (n = 11)	VBA complex was transposed using Teflon felts. In two cases, it was not possible to thoroughly mobilize the VBA away from the trigeminal nerve, and shredded Teflon felt was simply interposed between the nerve and vessel.	Efficacy; pain recurrence rate; postoperative complications rate.	Complete pain relief was achieved in 100% of cases. Four out of 13 patients reported pain recurrence at last follow-up; of these, 2 were treated with interposition; two patients were lost to follow-up; postoperative complications included: diplopia (2/13), ipsilateral limb ataxia (1/13).
Yu et al. [10]	Microvascular decompression by interposition method for treatment of trigeminal neuralgia due to vertebrobasilar dolichoectasia: a retrospective single-center study	2022	Case series	30 (21/9, 63.03 y.o.)	Interposition	A shredded Teflon sponge was placed in the neurovascular conflicting area between the VBA complex and the trigeminal nerve.	Efficacy; pain recurrence rate; postoperative complications rate.	Complete pain relief was achieved in 100% of cases; 10% of patients reported pain recurrence at the last follow-up; no postoperative complications were reported.
Shulev et al. [36]	Microvascular decompression in trigeminal neuralgia following vertebrobasilar dolichoectasia	2020	Case series	14 (4/10, 66 y.o.)	Interposition	A shredded Teflon sponge was placed in the neurovascular conflicting area between the VBA complex and the trigeminal nerve.	Efficacy; pain recurrence rate; postoperative complications rate.	Complete pain relief was achieved in 100% of cases; no patients reported pain recurrence at the last follow-up; postoperative complications were reported in 50% of patients and included: facial numbness (7%), abducens nerve dysfunction (7%), trochlear nerve dysfunction (7%), dysfunction of facial nerve (14%), hypoacusia (14%).
Apra et al. [3]	Microvascular decompression is an effective therapy for trigeminal neuralgia due to dolichoectatic basilar artery compression: case reports and literature review	2017	Case series	3 (3/0, 63.3 y.o.)	Interposition	Pieces of Dacron and Teflon were placed in the neurovascular conflicting area between the VBA complex and the trigeminal nerve.	Efficacy; pain recurrence rate; postoperative complications rate.	Complete pain relief was achieved in 100% of cases; no patients reported pain recurrence at the last follow-up; one out of 3 patients reported postoperative sixth nerve palsy with diplopia.
Sun et al. [50]	Clinical analysis and surgical treatment of trigeminal neuralgia caused by vertebrobasilar dolichoectasia: A retrospective study	2017	Case series	15 (8/7, 60.8 y.o.)	Interposition	Pieces of Teflon were placed in the neurovascular conflicting area between the VBA complex and the trigeminal nerve.	Efficacy; pain recurrence rate; postoperative complications rate.	Complete pain relief was achieved in 100% of cases; no patients reported pain recurrence at the last follow-up; postoperative facial numbness was reported in 15% of patients.
Ma et al. [30]	Clinical analysis of trigeminal neuralgia caused by vertebrobasilar dolichoectasia	2013	Case series	11 (8/3, 62.5 y.o.)	Interposition	Pieces of Teflon were placed in the neurovascular conflicting area between the VBA complex and the trigeminal nerve.	Efficacy; pain recurrence rate; postoperative complications rate.	Complete pain relief was achieved in 81% of cases, partial remission was reported in the remaining cases; no patients reported pain recurrence at the last follow-up; postoperative complications included: facial numbness (27%), facial palsy (9%), hemifacial spasm (9%).
El-Ghandour et al. [16]	Microvascular Decompression in the Treatment of Trigeminal Neuralgia Caused by Vertebrobasilar Ectasia	2010	Case series	10 (6/4, 54 y.o.)	Interposition	Pieces of Teflon were placed in the neurovascular conflicting area between the VBA complex and the trigeminal nerve.	Efficacy; pain recurrence rate; postoperative complications rate.	Complete pain relief was achieved in 80% of cases; no patients reported pain recurrence at the last follow-up; postoperative complications included: trochlear nerve paresis (10%) and mild facial weakness (10%)

y.o.: years old; VBA: vertebrobasilar artery.

## 4. Conclusions

Despite potential risks, MVD offers the best chance for long-term pain relief in patients suffering from VBD-induced TN. Future research should aim to further refine these surgical techniques and reduce the risk of postoperative complications, ultimately improving outcomes for this subset of patients.

## Figures and Tables

**Table 1:** Barrow Neurological Institute (BNI) pain intensity score.

Score	
I	No trigeminal pain, no medication
II	Occasional pain, not requiring medication
III	Some pain, adequately controlled with medication
IV	Some pain, not adequately controlled with medication
V	Severe pain or no pain relief

**Table 2:** Grade of compression of the Trigeminal Nerve.

Grade	
I	The vessel is simply in contact with the nerve but without any visible deformity of the root.
II	There is displacement or distortion of the root.
III	A clear-cut and marked indentation on the root is present.

## Data Availability

All the data generated in the present study have been included in this paper after anonymization.
